# Electropolymerization of a New Diketopyrrollopyrrole Derivative into Inherent Chiral Polymer Films

**DOI:** 10.3390/nano14221776

**Published:** 2024-11-05

**Authors:** Felix Niebisch, Ullrich Scherf, Alex Palma-Cando

**Affiliations:** 1 Department of Chemistry, Macromolecular Chemistry and Wuppertal Center for Smart Materials @ Systems (CM@S), Bergische Universität Wuppertal, Gaußstr. 20, 42119 Wuppertal, Germany; 2 Grupo de Investigación Aplicada en Materiales y Procesos (GIAMP), School of Chemical Sciences and Engineering, Yachay Tech University, Hda. San José s/n y Proyecto Yachay, Urcuqui 100115, Ecuador

**Keywords:** chiral conjugated polymers, electropolymerization, diketopyrrollopyrrole, 3,4-ethylenedioxythiophene, planar chirality, circular dichroism

## Abstract

Electropolymerization is a convenient way to obtain conducting polymers (CPs) directly adhered to an electrode surface. CPs are well-known for their various application fields in photovoltaic cells, chemical sensors, and electronics. By implementing chirality into a CP, the application possibilities will spread further onto chiral sensors or optoelectronics. In this work, we introduce a new inherently chiral polymer based on a macrocyclic 3,4-ethylenedioxythiophene-diketopyrrolopyrrole-3,4-ethylenedioxythiophene triad (EDOT-DPP-EDOT) fused by 1,4-phenylene groups, which was prepared via oxidative electropolymerization directly on the electrode surface. The investigation of the chiroptical properties was performed by circular dichroism spectroscopy in the solid state. The enantiomeric pure polymer films obtained showed dissymmetry factors of up to −2.71 × 10^−4^, whereby linear dichroism contributions can be widely excluded.

## 1. Introduction

Since the end of the 20th century, the electrochemical synthesis of conducting polymers (CPs) has been used as a versatile tool to deposit thin films of these generally hardly processable polymers [1]. The main advantage of this technique is the possibility of synthesizing CPs directly onto the electrode surface while obtaining it cleanly without any further work up [2]. Additionally, the thickness of the CPs can be controlled easily [3]. Polymer-coated electrodes can be used directly in electronic components as photovoltaic cells [4,5] and sensors [6,7,8]. In recent decades, research in the field of electrochemical CP synthesis has focused on the oxidative polymerization of heterocyclic compounds containing thiophene, pyrrole, anillin, or carbazole units [9,10,11]. A suitable oxidation potential, combined with the ability of the substrate to adhere and grow on a specific electrode surface, are crucial for obtaining feasible polymer films.

One extensively investigated CP is poly-3,4-ethylenedioxythiophene (PEDOT). Wet chemical synthesis mostly leads to black and insoluble polymers, whereas the low oxidation potential of EDOT enables electropolymerization, as reported for the first time in 1997 [12]. Electrodeposited PEDOT films combine noteworthy stability under ambient conditions, intrinsic conductivity, and the possibility of easily tailoring the properties of the formed polymers/composites. For example, the morphology of the films can be controlled by the number of potential sweep cycles, the solvent of choice, and using different polymerization potentials and electropolymerization methods, as already demonstrated [4,13,14]. Composites of electrodeposited PEDOT/polystyrene sulfonate (PSS) can be widely used in photovoltaic cells [4,5,14,15,16], OLEDs [17], and chemical sensors [6,18], only to mention a few. The research on further application areas is still ongoing today.

Diketopyrrolopyrrole (DPP) and its derivatives are also well-known structures for synthesizing CPs, and it belongs to a class of frequently used pigments. Small molecules, as well as polymers, exhibit properties such as high stability and low-cost synthesis [19]. Their potential application areas lie in organic photovoltaics [20,21], electronic components [22,23], or chemical sensors [24,25]. Processability, a widespread problem of DPP derivatives due to their solubility issues [26], can be improved by introducing alkyl side chains onto the lactam unit [27,28]. Additional flanking of the DPP core with heterocycles results in donor–acceptor molecules [27,29]. Due to its high oxidation potential, the pure DPP core cannot be electropolymerized; therefore, successful electropolymerizations have been reported for EDOT, bithiophene, carbazole, and naphthalene flanked DPP monomers [30,31,32]. The combination of DPP with EDOT is promising because of the low oxidation potential, high stability, and the formation of a conjugated backbone [30,31]. An example of electropolymerized EDOT-DPP-EDOT bearing linear alkyl sidechains has already been reported in the literature [30].

Introducing chirality in electropolymerizable monomers can extend the application potential of the resulting chiral conducting polymers (CCPs) onto optoelectronics [33] and chiral sensors [34]. In the literature to date, chirality in electropolymerized CCPs has mainly been introduced by the implementation of a chiral side chain [33,35,36], or, in rare cases, helices have been constructed using appropriate chiral molecules [37] or chiral inducers [38]. Planar chirality, on the other hand, is less common. To the best of our knowledge, no macrocyclic bridged compounds with planar chirality have been used in electropolymerizations, so this procedure will expand the range of possible structural motifs.

Herein, we present a new inherently chiral polymer film consisting of a planar chiral EDOT-DPP-EDOT triad, which exhibits dissymmetry factors of up to −2.71 × 10^−4^ in the solid state, directly measured for the polymer films on the ITO electrode.

## 2. Materials and Methods

### 2.1. Reagents and Experimental Methods

All reagents and dry solvents were obtained from commercial suppliers and were used without further purification. Chemical reactions were carried out in an argon atmosphere under dry conditions using a Schlenk line.

ALUGRAM^®^ SIL G/UV254 plates (Carl Roth GmbH + C. KG, Karlsruhe, Germany) were used for thin-layer chromatography. Chromatographic purifications were carried out using Flash-Chromatogrpahie-System Reveleris® X2 (BÜCHI Labortechnik, Essen, Germany) together with commercially available FlashPure ID cartridges (particle size: 35–45 µm, pore size: 53–80 Å) from the same company. Different eluent mixtures were used as indicated in the synthetic procedures at 25 °C, and substances were detected using implemented UV or ELSD detectors. ^1^H- and ^13^C-NMR as well as 2D spectra were measured on Bruker Avance 400 or Avance III 600 spectrometers (Bruker, Bremen, Germany) at 300 K. FD mass spectroscopy was conducted on a JEOL AccuTOF-GCX spectrometer (JEOL GmbH, Freising, Germany), while APCI mass spectroscopy was performed on a Bruker microTOF instrument.

For analytical HPLC-measurements, a Jasco 2000plus HPLC (Jasco Deutschland GmbH, Pfungstadt, Germany) system with a UV-2075 UV detector was utilized, along with a CHIRALPAK^®^IA column (4.6 × 250 mm, particle size: 5 µm) and a CHIRALPAK IA pre-column (4.6 × 250 mm, particle size: 5 µm) (DAICEL Corporation, Raunheim, Germany). Preparative separations were carried out using a Shimadzu Nexera Prep system (Shimadzu, Duisburg, Germany) with an SPD-40AV UV–vis detector, a CHIRALPAK^®^IA (20 × 250 mm, particle size: 5 µm), and a CHIRALPAK^®^IA pre-column (4.6 × 250 mm, particle size: 5 µm). 

IR spectra were obtained using a FT/IR-4700 spectrometer with an ATR PRO470-H unit (Jasco Deutschland GmbH). UV–vis spectra were recorded with a JASCO V-670 spectrometer (Jasco Deutschland GmbH) in THF at room temperature using a quartz cuvette (d = 10 mm) and an optical density of <1.0. Photoluminescence spectra were acquired using a Horiba Scientific FluroMax-4 spectrofluorometer (Horiba Europe GmbH, Oberursel, Germany) in THF with a quartz cuvette (d = 10 mm) and an optical density of <0.1.

Circular dichroism measurements were conducted at 25 °C using a JASCO J-810 spectropolarimeter equipped with a PTC-423S Peltier (Jasco Deutschland GmbH) and a 150 W Xenon bulb in both solution and solid phase. Solution-based investigations were performed in a quartz cuvette (d = 10 mm) in THF with an optical density of approximately 0.89. Solid-phase investigations were carried out with electrodeposited polymer film on ITO working electrodes using a custom-made carrier. To eliminate artifacts and linear dichroism, the polymer film was rotated about 90°and measured three times.

AFM measurements were carried out using diInnova (Bruker) with polymer films deposited on ITO working electrodes in intermitted mode. All measurements were performed under the same conditions. Two scans were performed on each sample. The thickness of the polymer films was determined by scratching the surface and then comparing the height of the center of the scratch with the polymer film in at least three different spots.

Optical rotation measurements were accomplished on a Krüss P8000-T (A. Krüss Optronic GmbH, Hamburg, Germany) polarimeter in dichloromethane at 20 °C.

### 2.2. Synthesis of Compound (−)/(+)-EDOT-DPP-EDOT

2-(2,3-Dihydrothien [3,4-b][1,4]dioxin-5-yl)-4,4,5,5-tetramethyl-1,3,2-dioxa-borolan (2) was prepared according to an adapted literature procedure with some modification [39]. In a three-necked round-bottom flask, 2,3-dihydrothieno [3,4-b][1,4]dioxine (1.00 g, 7.03 mmol) was dissolved in THF (15 mL) under an argon atmosphere. The mixture was cooled to −78 °C, and *n*BuLi in THF (496 mg, 4.84 mL, 1.6 molar, 1.1 eq., 7.74 mmol) was added dropwise using a syringe, and the temperature was slowly raised to 0 °C. After 30 min, the mixture was again cooled to −78 °C and 2-isopropoxy-4,4,5,5-tetramethyl-1,3,2-dioxaborolan (2.49 g, 2.73 mL, 1.9 eq., 13.4 mmol) was added dropwise to the solution. The reaction mixture was allowed to reach room temperature and stirred for an additional 16 h. After quenching with water, the mixture was extracted with dichloromethane and the combined organic phase was dried over MgSO_4_. The crude product was purified by flash column chromatography (eluent: cyclohexane/ethyl acetate, 100:0 → 90:10) and recrystallized from hexane to produce compound 2 as an off-white solid in a yield of 60% (1.13 g, 4.21 mmol). ^1^H-NMR (600 MHz, CDCl_3_): δ [ppm] = 6.62 (d, J = 0.9 Hz, 1H), 4.34–4.15 (m, 4H), 1.34 (d, J = 0.9 Hz, 12H). ^13^C{H}-NMR (151 MHz, CDCl_3_): δ [ppm] = 149.2, 142.4, 107.6, 83.9, 65.2, 64.4, 24.8. HRMS (APCI): *m*/*z* calcd: 291.0835; found: 291,0837.

3,6-bis(4-bromophenyl)-2,5-dihydropyrrolo [3,4-c]pyrrole-1,4-dione (Br-DPP) was prepared according to an adapted procedure published elsewhere [40]. Sodium *tert*-butoxide (13.7 g, 4.5 eq., 142 mmol) was placed in a three-necked round-bottom flask under argon atmosphere and was dissolved in *tert*-amyl alcohol (450 mL) at 80 °C for 1 h. Once all the sodium *tert*-butoxide had dissolved, 4-bromobenzontrile (17.2 g, 3.0 eq., 97.7 mmol) was added to the reaction and stirred at the same temperature for 1 h. Subsequently, diethyl succinate (5.50 g, 31.6 mmol, 5.25 mL) was added dropwise to the reaction mixture and the reaction was stirred at 105 °C for 16 h. The reaction mixture was cooled down to 50 °C and 30 mL methanol and 15 mL conc., aqueous hydrochloric acid was added. The precipitate was filtered off and the residue washed several times with hot methanol and hot water. The resultant solid was dried in a vacuum oven to obtain compound BR-DPP as a dark red solid in a yield of 51% (7.21 g, 16.2 mmol), which was used in the next step without further purification.

*(rac)-ansa*-3,6-bis(4-bromophenyl)-2,5-dihydropyrrolo [3,4-c]pyrrole-1,4-dione (*(rac)*-Br-DPP) was prepared following a known procedure in the literature [41]. In a three-necked round-bottom flask, compound Br-DPP (1.63 g, 3.66 mmol) and potassium carbonate (2.53 g, 5.0 eq., 18.3 mmol) were dissolved in dry DMF (145 mL) under an argon atmosphere. The reaction mixture was stirred at 120 °C for 30 min and then cooled down to 80 °C. Subsequently, 1,12-dibromododecane (3.00 g, 2.5 eq., 9.15 mmol) was added, and the mixture was stirred at 80 °C for 16 h. The mixture was poured into an ice/water mixture and the precipitate was filtered off using a Buchner funnel. The residue was collected with copious amount of chloroform and then purified via flash column chromatography (eluent: dichloromethane). After recrystallization from a mixture of hexane and chloroform, compound *(rac)*-Br-DPP was obtained as red crystals in an 8% yield (171 µg, 279 µmol). ^1^H-NMR (400 MHz, CDCl_3_): δ [ppm] = 7.70–7.56 (m, 8H), 4.30–3.58 (m, 4H), 1.29–1.02 (m, 20H). ^13^C{H}-NMR (151 MHz, CDCl_3_): δ [ppm] = 163.3, 148.0, 132.3, 130.1, 127.4, 125.9, 110.8, 41.8, 28.6, 27.8, 27.8, 27.7, 27.0, 25.3. HRMS (FD): *m*/*z* calcd: 610.0867; found: 610.0831.

*(rac)*-EDOT-DPP-EDOT was prepared by placing the following reagents in a microwave vessel: *(rac)*-Br-DPP (250 mg, 408 µmol), compound 2 (175 mg, 4.0 eq., 653 µmol), potassium carbonate (226 mg, 4.0 eq., 1.63 mmol), and Pd(PPh_3_)_4_ (47.2 mg, 10 mol%, 40.8 µmol), dissolved in toluene (11.3 mL) and degassed water (2.83 mL) under an argon atmosphere. Additionally, a drop of Aliquat 336 was added and the reaction mixture was heated at 115 °C for 16 h. Afterwards, water was added to the reaction mixture and the aqueous phase was extracted with chloroform. The combined organic phases were dried over MgSO_4_, and the crude product was purified by flash column chromatography (eluent: dichloromethane/methanol 98:2) and recrystallized from chloroform and hexane to obtain compound *(rac)*-EDOT-DPP-EDOT as a dark violet solid in a yield of 47% (141 mg, 192 µmol). ^1^H-NMR (400 MHz, CDCl_3_): δ [ppm] = 7.87 (s, 8H), 6.37 (d, J = 1.8 Hz, 2H), 4.41–4.21 (m, 10H), 3.82–3.72 (m, 2H), 1.36–1.04 (m, 20H). ^13^C{H}-NMR (151 MHz, CDCl_3_): δ [ppm] = 163.7, 148.3, 142.5, 139.6, 136.0, 129.0, 126.6, 126.0, 116.9, 110.9, 99.3, 65.0, 64.5, 42.1, 28.7, 28.0, 27.8, 27.7, 25.4. HRMS (FD): *m*/*z* calcd: 734,2517; found: 734,2484. UV–vis (THF): λ_max_ [nm] = 269, 324, 380, 511. PL (THF, λ_exc._ [nm] = 500): λ_max_ [nm] = 583, 624, 697.

Enantiopure *EDOT-DPP-EDOT ((−)*-*EDOT-DPP-EDOT*, *(+)*-*EDOT-DPP-EDOT)*

A solution of the racemic mixture with concentrations of 0.5 mg/mL for analytical purposes and 5 mg/mL for preparative purposes was injected with volumes of 0.5 µL and 1 mL, respectively. Analytical separations were performed at 40 °C in hexane/chloroform (60/40) as eluent, and preparative separations at 39 °C in hexane/chloroform (50/50) as eluent.

Analytical HPLC (CHIRALPAK IA in hexane/chloroform = 60/40, 0.5 mL/min, 40 °C, λ = 254 nm): t_R_ *(−)* = 18.6 min, ≥99.9%ee t_R_ *(+)* = 33.2 min, ≥99.9%ee, R_S_ = 11.2.

The racemic mixture compound *(rac)*-EDOT-DPP-EDOT was separated by preparative HPLC (CHRIALPAK IA in hexane/chloroform = 50/50, 16 mL/min, 39 °C, λ = 510 nm, injection amount 5 mg), to yield the enantiopure compounds as dark solids.

*(−)*-*EDOT-DPP-EDOT* (CHIRALPAK IA in (hexane/chloroform = 50/50, 16 mL/min, 39 °C, λ = 510 nm): t_R_ = 13.1 min, ≥99.9%ee. ${\left[\alpha \right]}_{D}^{20}$ [°∙mL∙dm^−1^∙g^−1^] = −572 (conc. = 0.25 mg/mL, DCM), ${\left[\alpha_{m}\right]}_{D}^{20}$ [°∙cm^2^∙dmol^−1^] = −4204.

*(+)*-*EDOT-DPP-EDOT* (CHIRALPAK IA in (hexane/chloroform = 50/50, 16 mL/min, 39 °C, λ = 510 nm): t_R_ = 21.9 min, ≥99.9%ee. ${\left[\alpha \right]}_{D}^{20}$ [°∙mL∙dm^−1^∙g^−1^] = 564 (conc. = 0.25 mg/mL, DCM), ${\left[\alpha_{m}\right]}_{D}^{20}$ [°∙cm^2^∙dmol^−1^] = 4145.

### 2.3. Electrochemical Methods

For electrochemical polymerization and characterization, the potentiostat/galvanostat PAR VersaSTAT 4 was used in combination with its controller program VerstaStudio 4.0. For polymerization, a 1.0 mM solution of racemic or enantiopure EDOT-DPP-EDOT monomers together with 0.1 M TBAPF_6_ as a supporting electrolyte was placed in a 10 mL three-electrode cell and dissolved in 10 mL of dichloromethane under an argon atmosphere at 25 °C. A platinum disc electrode (Pt, 1 mm diameter) or ITO on glass (~1.5 × 1.2 cm) was used as working electrodes (WEs), platinum wire as the counter electrode (CE), and Ag^0^/AgNO_3_ (0.1 M AgNO_3_, 0.1 M TBAP in acetonitrile, 0.56 V vs. NHE) as the reference electrode (RE).

Thin polymer films were deposited on Pt-WE by performing 10 cycles between −0.05 V and 1.0 V with a scan rate of 0.1 Vs^−1^. Stability and adherence characterizations were carried out in a monomer-free dichloromethane solution with TBAPF_6_ as a supporting electrolyte. The polymer films were subjected to 20 cycles between 0.05 V and 0.65 V with a scan rate of 0.2 Vs^−1^ followed by 2 additional cycles at different scan rates from 0.2 Vs^−1^ to 0.005 Vs^−1^. 

Thin racemic and enantiopure polymer films for AFM measurements were potentiodynamically generated on ITO-WE by running 10 cycles between −0.05 V and 1.0 V at a scan rate of 0.1 Vs^−1^. Subsequently, the polymer film was carefully washed with acetonitrile/dichloromethane and dried in ambient conditions.

Thick racemic and enantiopure polymer films for optical measurements were potentiostatically produced on ITO-WE by applying a potential of 0.9 V for 10 min followed by a potential of 0.0 V for 60 s. The polymer film was then carefully washed with acetonitrile/dichloromethane and dried under ambient conditions.

## 3. Results and Discussion

**Scheme 1:**
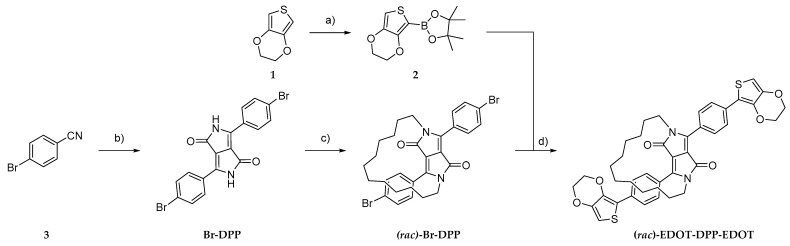
Synthesis of *(rac)*-EDOT-DPP-EDOT: (**a**) *n*BuLi, isopropoxy boronic acid pinacol ester, THF, −78 °C → 0 °C, 16 h; (**b**) I: *t*-BuONa, *t*-amyl alcohole, 80 °C, 1 h; II: diethyl succinate, *t*-amyl alcohol, 105 °C, 16 h; (**c**) I: K_2_CO_3_, DMF, 120 °C; II: 1,12-dibromododecane, 80 °C, 16 h; (**d**) K_2_CO_3_, Pd(PPh_3_)_4_, Aliquat 336, Tol/H_2_O, 115 °C, 16 h.

The synthesis route to the *(rac)*-EDOT-DPP-EDOT monomer is depicted in Scheme 1. The borylated 3,4-ethylenedioxythiophene (2) was obtained by treating 3,4-ethylenedioxythiophene with *n*-butyllithium and a subsequent addition of isopropoxy boronic acid pinacol ester, as described in the literature [39]. The dibrominated diketopyrrollopyrolle unit (Br-DPP) was prepared according to a literature-adapted procedure via the succinic ester route and used in the next step without further purification [40,42]. Planar chirality was introduced by installing a C_12_-carbon bridge through deprotonation of nitrogens with potassium carbonate and subsequent addition of 1,12-dibromododecane to obtain *(rac)*-Br-DPP [41]. Using the well-known Ruggli–Ziegler dilution principle, dimerization/polymerization was attempted to be suppressed in this step [43]. Successful incorporation of the bridge was proved by FD mass spectroscopy, which ruled out dimers or polymers. The racemic monomer *(rac)*-EDOT-DPP-EDOT was prepared upon Suzuki–Miyaura cross-coupling of borylated EDOT (2) and previously obtained *(rac)*-Br-DPP. The reaction was carried out in toluene/water using Pd(PPh_3_) as the catalyst and potassium carbonate as the base. All the products could be clearly identified by NMR and mass spectrometry and were obtained in excellent purity (see Figures S1–S3).

**Figure 1:**
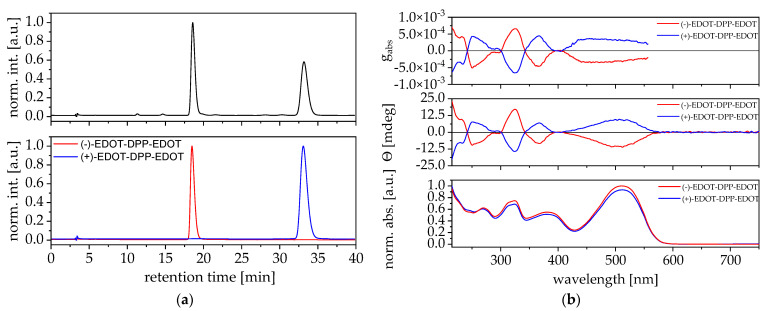
(**a**) HPLC separation of *(rac)*-EDOT-DPP-EDOT. (**b**) Dissymmetry factors g_abs_, ellipticities Θ, and absorption spectra of both pure enantiomers.

The appropriate conditions for preparative HPLC enantiomer separation were at first determined by analytical HPLC measurements. The elugrams are shown in Figure 1a. The analytical measurement was carried out at 40 °C with amylose as the stationary phase and hexane/chloroform (60:40) as the eluent. The applied conditions resulted in a resolution value of 11.2 as a measure of the extent of separation between the enantiomer peaks at different retention times.

Apart from the eluent composition change to 50:50, the conditions have been adopted for the preparative separation. Both enantiomers were obtained in 99.9% enantiomeric excess; the purity was determined using analytical HPLC and the integrated peak areas.

The chiroptical properties of the pure enantiomers were measured in a solution with THF as the solvent. The dissymmetry factors g_abs_, ellipticities, and the absorption spectra of the monomers are shown in Figure 1b. A mirror symmetry of the recorded CD spectra is recognizable, which clearly proves the presence of both enantiomers [44]. The CD spectra show monosignate Cotton effects over the entire absorption range for the respective peak maxima. The most bathochromically shifted absorbance signal at 511 nm can be assigned to the conjugated π-electron system of the monomer [45]; for this maximum, the measured dissymmetry factors g_abs_ are +3.12 × 10^−4^ and −3.20 × 10^−4^, respectively.

Electrochemical studies of *(rac)*-EDOT-DPP-EDOT were performed in a three-electrode cell using a platinum WE, platinum as the CE, and a silver/silver nitrate RE. Polymerization was performed in dry dichloromethane under an argon atmosphere with TBAPF_6_ as a supporting electrolyte. Further details are described in the experimental part.

**Figure 2:**
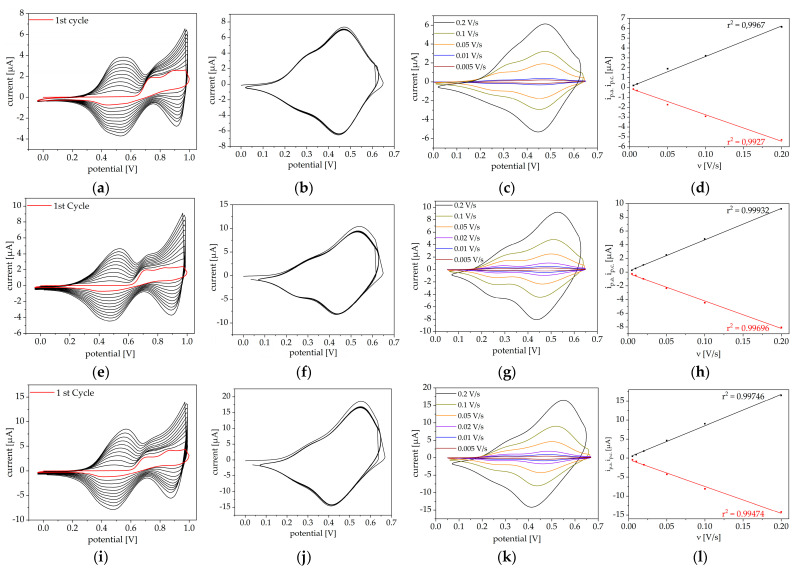
Electropolymerization of (**a**) *(rac)*-EDOT-DPP-EDOT, (**e**) *(−)*-EDOT-DPP-EDOT, and (**i**) *(+)*-EDOT-DPP-EDOT: Pt disc-WE, 1.0 mM monomer, and 0.1 M TBAPF_6_ in DCM, −0.05–1.0 V vs. Ag^0^/AgNO_3_, 0.1 Vs^−1^, 10 cycles, 1st cycle red. Stability measurement of (**b**) P-*(rac)*-EDOT-DPP-EDOT/Pt, (**f**) P-*(−)*-EDOT-DPP-EDOT/Pt, and (**j**) P-*(+)*-EDOT-DPP-EDOT/Pt: 0.1 M TBAPF_6_ in ACN, 0.05–0.65 V vs. Ag^0^/AgNO_3_, 0.2 Vs^−1^, 20 cycles. Adherence measurement of (**c**) P-*(rac)*-EDOT-DPP-EDOT/Pt, (**g**) P-*(−)*-EDOT-DPP-EDOT/Pt, and (**k**) P-*(+)*-EDOT-DPP-EDOT/Pt: 0.1 M TBAPF_6_ in ACN, 0.05–0.65 V vs. Ag^0^/AgNO_3_, 0.2 Vs^−1^–0.005 Vs^−1^, 2 cycles. Peak current plotted as a function of the scan rate for the (**d**) P-*(rac)*-EDOT-DPP-EDOT/Pt, (**h**) P-*(−)*-EDOT-DPP-EDOT/Pt, and (**l**) P-*(+)*-EDOT-DPP-EDOT/Pt electrodes.

First, the oxidation peak potentials of the *(rac)*-monomer were determined using cyclic voltammetry, which are located at 0.75 V and 0.90 V (see Figure 2a). Polymerization between −0.05 and 1.00 V under potentiodynamic conditions led to the desired polymer film, adhering to the electrode surface. The cyclic voltammogram is shown in Figure 2a. After the first cycle and reaching the peak potential at 0.90 V, a well-developed reversible redox peak appears at approximately 0.50 V, which can be assigned to the charging and discharging of the formed polymer adhering to the electrode surface. Additionally, the increase in the baseline current in proportion to the number of cycles leads to the conclusion of a progressive formation of an electrically conducting polymer.

The polymer film obtained shows high stability (see Figure 2b) in the range of 0.05 V to 0.65 V over 20 cycles in a monomer-free acetonitrile solution in the presence of the TBAPF_6_ electrolyte. Additionally, a linear dependency between the scan rate and peak current (see Figure 2c,d) can be observed, which indicates good adhesion of the polymer film towards the electrode surface as well as an effective charge transportation through the layers [46]. Similar results are obtained for polymer films composed of the enantiopure *(−)*- and *(+)*-monomers (Figure 2e–l).

**Figure 3:**
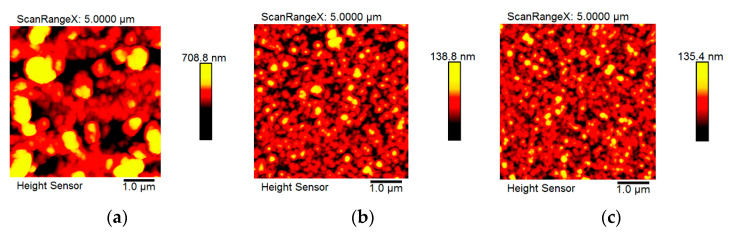
Tapping mode AFM images of polymer films on ITO. (**a**) P-(rac)-EDOT-DPP-EDOT; (**b**) P-*(−)*-EDOT-DPP-EDOT; (**c**) P-*(+)*-EDOT-DPP-EDOT. The films were prepared potentiodynamically from −0.05–1.0 V from a 1.0 mM solution of the monomers in dichloromethane using 0.1 M TBAPF_6_ as a supporting electrolyte (for corresponding 3D images, see Figure S4).

The AFM morphology analysis of the films shows the typical cauliflower-like morphology of electrochemically synthesized polymers (see Figure 3). Interestingly, when comparing the average thickness values of the polymer films produced, the polymer film from the racemic monomer is approximately four times thicker than the polymer films from the enantiomerically pure monomers (see Table 1). A similar correlation can be observed for the roughness of the films with cauliflower-like features of much smaller diameter for the films created from both enantiopure monomers. Based on these data, it can be interpreted that the stereoregular films from the enantiomeric pure monomers lead to a much denser packing in the solid state than the polymer films from the racemic monomer, resulting in these large differences. To ensure good comparability of the morphology, layer thicknesses, and roughnesses, all the polymers were synthesized on ITO electrodes and analyzed under the same conditions.

To investigate the chiroptical properties, thicker polymer films are required. For this reason, the polymer films for these measurements were produced potentiostatically by applying a voltage of 0.9 V for 10 min.

**Figure 4:**
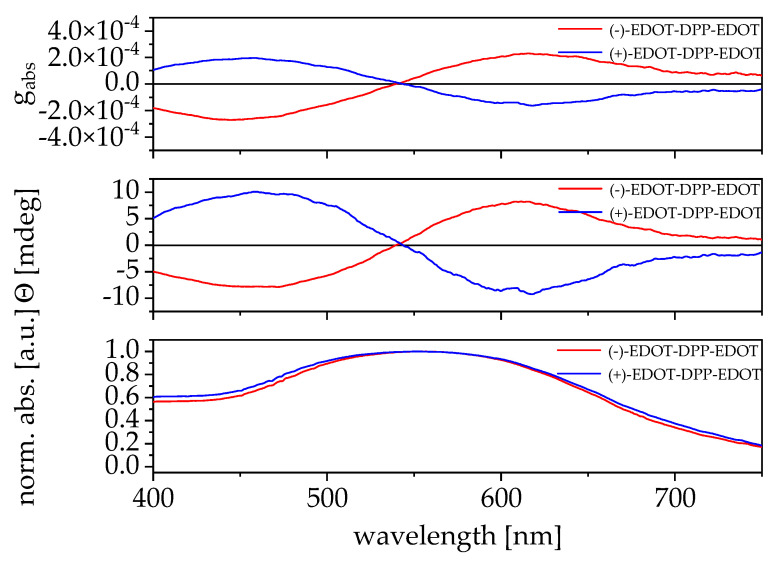
(**Top**) Dissymmetry factors g_abs_, (**mid**) ellipticities Θ, and (**bottom**) absorption spectra of both enantiopure polymer films on ITO, measured on a Jasco J-810 CD-spectropolarimeter (Jasco Deutschland GmbH).

If compared to the monomer spectra, a bathochromic shift in the longest wavelength maximum to 551 nm is visible, which can be attributed to the expansion of the π-electron system during the polymerization (see Figure 4). All three polymer films display very similar UV–vis spectra (see Figure S5). The CD spectra of the chiral polymer films show the occurrence of a bisignate Cotton effect due to the coupling of the planar chiral subunits with g_abs_ values approaching −2.71 × 10^−4^ for P-*(−)*-EDOT-DPP-EDOT and 1.63 × 10^−4^ for P-*(+)*-EDOT-DPP-EDOT and a zero crossing near the absorption maximum at 542 nm.

## 4. Conclusions

We successfully synthesized and separated new enantiopure macrocyclic bridged EDOT-DPP-EDOT triads linked by 1,4-phenylene groups with planar chirality. The inherently chiral polymers were generated via the oxidative electropolymerization of the corresponding EDOT-DPP-EDOT monomers. The obtained polymer films showed good stability and adherence to the electrode surface. The films from the racemic monomer mixture were approximately four times rougher and thicker than the films from the enantiopure monomers, thus demonstrating a denser solid-state packing of the polymer films created from the enantiopure monomer triads. The chiroptical properties of the polymer films were investigated in the solid state on ITO substrates. The polymer films from the enantiopure monomers show dissymmetry factors of −2.71 × 10^−4^ and 1.63 × 10^−4^ of the lowest energy lobes for P-*(−)*-EDOT-DPP-EDOT and P-*(+)*-EDOT-DPP-EDOT, respectively, ruling out linear dichroism contributions. The results introduce possibilities for chiroptical applications, e.g., in thin-film sensors.

## Figures and Tables

**Table 1:** Average roughnesses and thicknesses of electrogenerated polymer films on ITO.

Polymer	Roughness [nm]	Average Thickness [nm]
P-*(rac)*-EDOT-DPP-EDOT	105	~990
P-*(−)*-EDOT-DPP-EDOT	18	~280
P-*(+)*-EDOT-DPP-EDOT	18	~265

## Data Availability

The original contributions presented in the study are included in the article/Supplementary Materials; further inquiries can be directed to the corresponding author(s).

## Supplementary Materials

The following supporting information can be downloaded at https://www.mdpi.com/article/10.3390/nano14221776/s1. Figure S1: (a) ^1^H-NMR (600 MHz, CDCl_3_) of compound 2. (b) ^13^C{H}-NMR (151 MHz, CDCl_3_) of compound 2. Figure S2: (a) ^1^H-NMR (400 MHz, CDCl_3_) of *(rac)*-Br-DPP. (b) ^13^C{H}-NMR (101 MHz, CDCl_3_) of *(rac)*-Br-DPP. Figure S3: (a) ^1^H-NMR (400 MHz, CDCl_3_) of *(rac)*-EDOT-DPP-EDOT. (b) ^13^C{H}-NMR (101 MHz, CDCl_3_) of *(rac)*-EDOT-DPP-EDOT. Figure S4: Tapping mode AFM *3D* images of polymer films on ITO. (a) P-(rac)-EDOT-DPP-EDOT; (b) P-(−)-EDOT-DPP-EDOT; (c) P-(+)-EDOT-DPP-EDOT. Figure S5: UV–vis spectra of polymer films measured on ITO. P-*(rac)*-EDOT-DPP-EDOT measured on a JASCO V-670 spectrometer. P-*(−)*-EDOT-DPP-EDOT and P-*(+)*-EDOT-DPP-EDOT measured on a Jasco J-810 CD- spectropolarimeter.

## References

[1] Cosnier S., Karyakin A. (2010). Electropolymerization: Concepts, Materials and Applications.

[2] Frontana Uribe B.A., Palma-Cando A. (2022). Conducting Polymers. Kirk-Othmer Encyclopedia of Chemical Technology.

[3] Rendón-Enríquez I., Palma-Cando A., Körber F., Niebisch F., Forster M., Tausch M.W., Scherf U. (2023). Thin Polymer Films by Oxidative or Reductive Electropolymerization and Their Application in Electrochromic Windows and Thin-Film Sensors. Molecules.

[4] Erazo E.A., Ortiz P., Cortés M.T. (2023). Tailoring the PEDOT:PSS Hole Transport Layer by Electrodeposition Method to Improve Perovskite Solar Cells. Electrochim. Acta.

[5] Saito Y., Kitamura T., Wada Y., Yanagida S. (2002). Application of Poly(3,4-Ethylenedioxythiophene) to Counter Electrode in Dye-Sensitized Solar Cells. Chem. Lett..

[6] Ko S.H., Kim S.W., Lee Y.J. (2021). Flexible Sensor with Electrophoretic Polymerized Graphene Oxide/PEDOT:PSS Composite for Voltammetric Determination of Dopamine Concentration. Sci. Rep..

[7] Palma-Cando A., Scherf U. (2015). Electrogenerated Thin Films of Microporous Polymer Networks with Remarkably Increased Electrochemical Response to Nitroaromatic Analytes. ACS Appl. Mater. Interfaces.

[8] Räupke A., Palma-Cando A., Shkura E., Teckhausen P., Polywka A., Görrn P., Scherf U., Riedl T. (2016). Highly Sensitive Gas-Phase Explosive Detection by Luminescent Microporous Polymer Networks. Sci. Rep..

[9] Quiroz-Arturo H., Reinoso C., Scherf U., Palma-Cando A. (2024). Microporous Polymer-Modified Glassy Carbon Electrodes for the Electrochemical Detection of Metronidazole: Experimental and Theoretical Insights. Nanomaterials.

[10] Ibanez J.G., Rincón M.E., Gutierrez-Granados S., Chahma M., Jaramillo-Quintero O.A., Frontana-Uribe B.A. (2018). Conducting Polymers in the Fields of Energy, Environmental Remediation, and Chemical-Chiral Sensors. Chem. Rev..

[11] Palma-Cando A., Brunklaus G., Scherf U. (2015). Thiophene-Based Microporous Polymer Networks via Chemical or Electrochemical Oxidative Coupling. Macromolecules.

[12] Sotzing G.A., Reynolds J.R., Steel P.J. (1997). Poly(3,4-Ethylenedioxythiophene) (PEDOT) Prepared via Electrochemical Polymerization of EDOT, 2,2′-Bis(3,4-Ethylenedioxythiophene) (BiEDOT), and Their TMS Derivatives. Adv. Mater..

[13] Palma-Cando A.U., Frontana-Uribe B.A., Maldonado J.L., Hernández M.R. (2014). Control of Thickness of PEDOT Electrodeposits on Glass/ITO Electrodes from Organic Solutions and Its Use as Anode in Organic Solar Cells. Procedia Chem..

[14] Nasybulin E., Wei S., Kymissis I., Levon K. (2012). Effect of Solubilizing Agent on Properties of Poly(3,4-Ethylenedioxythiophene) (PEDOT) Electrodeposited from Aqueous Solution. Electrochim. Acta.

[15] Yan J., Sun C., Tan F., Hu X., Chen P., Qu S., Zhou S., Xu J. (2010). Electropolymerized Poly(3,4-Ethylenedioxythiophene):Poly(Styrene Sulfonate) (PEDOT:PSS) Film on ITO Glass and Its Application in Photovoltaic Device. Sol. Energy Mater. Sol. Cells.

[16] Terán-Alcocer Á., Bravo-Plascencia F., Cevallos-Morillo C., Palma-Cando A. (2021). Electrochemical Sensors Based on Conducting Polymers for the Aqueous Detection of Biologically Relevant Molecules. Nanomaterials.

[17] Saghaei J., Koodalingam M., Burn P.L., Gentle I.R., Pivrikas A., Shaw P.E. (2022). Effect of PEDOT:PSS on the Performance of Solution-Processed Blue Phosphorescent Organic Light-Emitting Diodes with an Exciplex Host. Mater. Adv..

[18] Wang Q., Sun H., Liu Q., Li L., Kong J. (2020). Electrodeposition of Three-Dimensional Network Nanostructure PEDOT/PANI for Simultaneous Voltammetric Detection of Ascorbic Acid, Dopamine and Uric Acid. ChemistrySelect.

[19] David J., Weiter M., Vala M., Vyňuchal J., Kučerík J. (2011). Stability and Structural Aspects of Diketopyrrolopyrrole Pigment and Its N-Alkyl Derivatives. Dyes Pigment..

[20] Gao K., Jo S.B., Shi X., Nian L., Zhang M., Kan Y., Lin F., Kan B., Xu B., Rong Q. (2019). Over 12% Efficiency Nonfullerene All-Small-Molecule Organic Solar Cells with Sequentially Evolved Multilength Scale Morphologies. Adv. Mater..

[21] Wienk M.M., Turbiez M., Gilot J., Janssen R.A.J. (2008). Narrow-Bandgap Diketo-Pyrrolo-Pyrrole Polymer Solar Cells: The Effect of Processing on the Performance. Adv. Mater..

[22] Ji Y., Xiao C., Wang Q., Zhang J., Li C., Wu Y., Wei Z., Zhan X., Hu W., Wang Z. (2016). Asymmetric Diketopyrrolopyrrole Conjugated Polymers for Field-Effect Transistors and Polymer Solar Cells Processed from a Nonchlorinated Solvent. Adv. Mater..

[23] Liu Z., Hu Y., Li P., Wen J., He J., Gao X. (2020). Enhancement of the Thermoelectric Performance of DPP Based Polymers by Introducing One 3,4-Ethylenedioxythiophene Electron-Rich Building Block. J. Mater. Chem. C Mater..

[24] Zhang H., Yang K., Chen C., Wang Y., Zhang Z., Tang L., Sun Q., Xue S., Yang W. (2018). 1,4-Diketo-Pyrrolo[3,4-c]Pyrroles (DPPs) Based Insoluble Polymer Films with Lactam Hydrogens as Renewable Fluoride Anion Chemosensor. Polymer.

[25] Jia L., Hao J., Wang S., Yang L., Liu K. (2023). Sensitive Detection of 4-Nitrophenol Based on Pyridine Diketopyrrolopyrrole-Functionalized Graphene Oxide Direct Electrochemical Sensor. RSC Adv..

[26] Leenaers P.J., Wienk M.M., Janssen R.A.J. (2020). Structural Design of Asymmetric Diketopyrrolopyrrole Polymers for Organic Solar Cells Processed from a Non-Halogenated Solvent. Org. Electron..

[27] Naik M.A., Venkatramaiah N., Kanimozhi C., Patil S. (2012). Influence of Side-Chain on Structural Order and Photophysical Properties in Thiophene Based Diketopyrrolopyrroles: A Systematic Study. J. Phys. Chem. C.

[28] Lange G., Tieke B. (1999). New Deeply Coloured and Fluorescent Polymers with 1,4-Dioxo-3,6-Diphenylpyrrolo[3,4-c]Pyrrole Units in the Main Chain. Macromol. Chem. Phys..

[29] Dhar J., Venkatramaiah N., Anitha A., Patil S. (2014). Photophysical, Electrochemical and Solid State Properties of Diketopyrrolopyrrole Based Molecular Materials: Importance of the Donor Group. J. Mater. Chem. C Mater..

[30] Zhu Y., Zhang K., Tieke B. (2009). Electrochemical Polymerization. of Bis(3,4-Ethylenedioxythiophene)- Substituted 1,4-Diketo-3,6-Diphenyl-Pyrrolo[3,4-c]Pyrrole (DPP) Derivative. Macromol. Chem. Phys..

[31] Zhu Y. (2006). New Diketopyrrolopyrrole(DPP)-Based Conjugated Polymers Prepared upon Palladium Catalyzed Polymerization and Electropolymerization Reactions. Ph.D. Thesis.

[32] Ponnappa S.P., Liu Q., Umer M., MacLeod J., Jickson J., Ayoko G., Shiddiky M.J.A., O’Mullane A.P., Sonar P. (2019). Naphthalene Flanked Diketopyrrolopyrrole: A New Conjugated Building Block with Hexyl or Octyl Alkyl Side Chains for Electropolymerization Studies and Its Biosensor Applications. Polym. Chem..

[33] Han H., Choi J.H., Ahn J., Lee H., Choi C., Jung W., Yeom J., Hwang D.K., Sung B.J., Lim J.A. (2023). Chiral Diketopyrrolopyrrole-Based Conjugated Polymers with Intramolecular Rotation–Isomeric Conformation Asymmetry for Near-Infrared Circularly Polarized Light-Sensing Organic Phototransistors. ACS Appl. Mater. Interfaces.

[34] Dong L., Zhang Y., Duan X., Zhu X., Sun H., Xu J. (2017). Chiral PEDOT-Based Enantioselective Electrode Modification Material for Chiral Electrochemical Sensing: Mechanism and Model of Chiral Recognition. Anal. Chem..

[35] Albano G., Aronica L.A., Pescitelli G., Di Bari L. (2024). Chiral Diketopyrrolo[3,4-c]Pyrrole-Based Oligothiophenes: Synthesis and Characterization of Aggregated States in Solution and Thin Films. Chirality.

[36] Otaki M., Komaba K., Goto H. (2022). Synthesis of Polythiophene-Based Chiral Magnetic Block Copolymers with High Stereoregularity. ACS Appl. Polym. Mater..

[37] Vacek J., Hrbáč J., Strašák T., Církva V., Sýkora J., Fekete L., Pokorný J., Bulíř J., Hromadová M., Crassous J. (2018). Anodic Deposition of Enantiopure Hexahelicene Layers. ChemElectroChem.

[38] Goto H., Akagi K. (2004). Preparation of Poly(3,4-Ethylenedioxythiophene) in a Chiral Nematic Liquid-Crystal Field. Macromol. Rapid Commun..

[39] Hassan Omar O., La Gatta S., Tangorra R.R., Milano F., Ragni R., Operamolla A., Argazzi R., Chiorboli C., Agostiano A., Trotta M. (2016). Synthetic Antenna Functioning as Light Harvester in the Whole Visible Region for Enhanced Hybrid Photosynthetic Reaction Centers. Bioconjug. Chem..

[40] Zhu H., Huang W., Huang Y., Yang J., Wang W. (2016). Narrow Band-Gap Donor-Acceptor Copolymers Based on Diketopyrrolopyrrole and Diphenylethene: Synthesis, Characterization and Application in Field Effect Transistor. Dyes Pigment..

[41] Yang Q., Zhang J., Wang L. (2022). Packing-Dependent Polymorphism: A Stimuli-Responsive Macrocyclic Diketopyrrolopyrrole. Dyes Pigment..

[42] Iqbal A., Jost M., Kirchmayr R., Pfenninger J., Rochat A. (1988). The synthesis and properties of 1,4-diketo-pyrrolo[3,4-c]pyrroles. Bull. Des Sociétés Chim. Belg..

[43] Ruggli P. (1912). Über Einen Ring Mit Dreifacher Bindung. Justus Liebigs Ann. Chem..

[44] Berova N., Di Bari L., Pescitelli G. (2007). Application of Electronic Circular Dichroism in Configurational and Conformational Analysis of Organic Compounds. Chem. Soc. Rev..

[45] Kirkus M., Wang L., Mothy S., Beljonne D., Cornil J., Janssen R.A.J., Meskers S.C.J. (2012). Optical Properties of Oligothiophene Substituted Diketopyrrolopyrrole Derivatives in the Solid Phase: Joint J- and H-Type Aggregation. J. Phys. Chem. A.

[46] Chen W.-C., Wen T.-C., Gopalan A. (2001). Electrochemical and Spectroelectrochemical Evidences for Copolymer Formation Between 2-Aminodiphenylamine and Aniline. J. Electrochem. Soc..

