# Supplementary material for: Electropolymerization of a New Diketopyrrollopyrrole Derivative into Inherent Chiral Polymer Films
Source: Nanomaterials (Basel). 2024 Nov 5;14(22):1776. doi: 10.3390/nano14221776 (PMC11597823; doi:10.3390/nano14221776)
Supplement: Supplementary file 1 [file nanomaterials-14-01776-s001.zip › nanomaterials-3271346-supplementary.pdf]

Supplementary Materials

Electropolymerization of a new Diketopyrrollopyrrole Derivative into Inherent Chiral Polymer Films

Felix Niebisch [1], Ullrich Scherf [1,*], Alex Palma-Cando [2,*]

[1] Department of Chemistry, Macromolecular Chemistry and Wuppertal Center for Smart Materials @ Systems (CM@S), Bergische Universität Wuppertal, Gaußstr. 20, 42119 Wuppertal, Germany
[2] Grupo de Investigación Aplicada en Materiales y Procesos (GIAMP), School of Chemical Sciences and Engineering, Yachay Tech University, Hda. San José s/n y Proyecto Yachay, Urcuqui 100115, Ecuador
*Correspondence: scherf@uni-wuppertal.de (U.S.); apalma@yachaytech.edu.ec (A.P.-C.)

Table of Content

^{1}H-and ^{13}C{H}-NMR spectra of compound **2** 2

^{1}H-and ^{13}C{H}-NMR spectra of compound ***(rac)*-Br-DPP** 3

^{1}H-and ^{13}C{H}-NMR spectra of compound ***(rac)*-EDOT-DPP-EDOT** 4

AFM tapping mode 3-D images 5

UV/Vis spectra of the polymer films on ITO 5

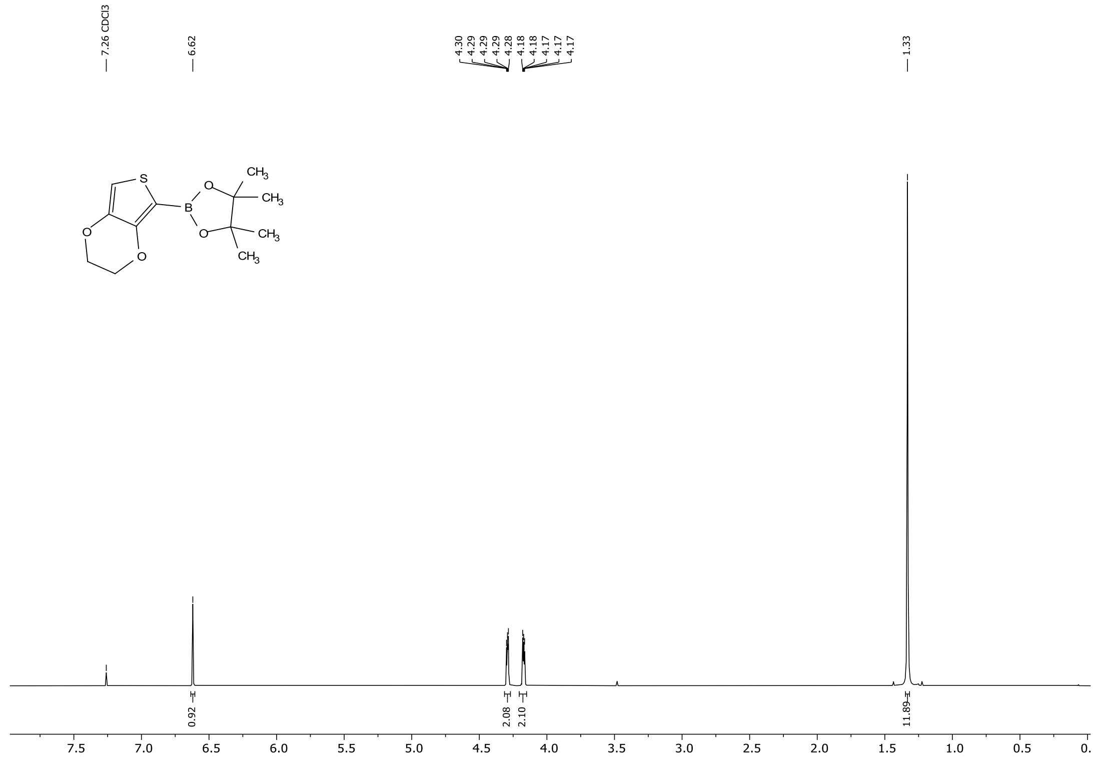

(a)

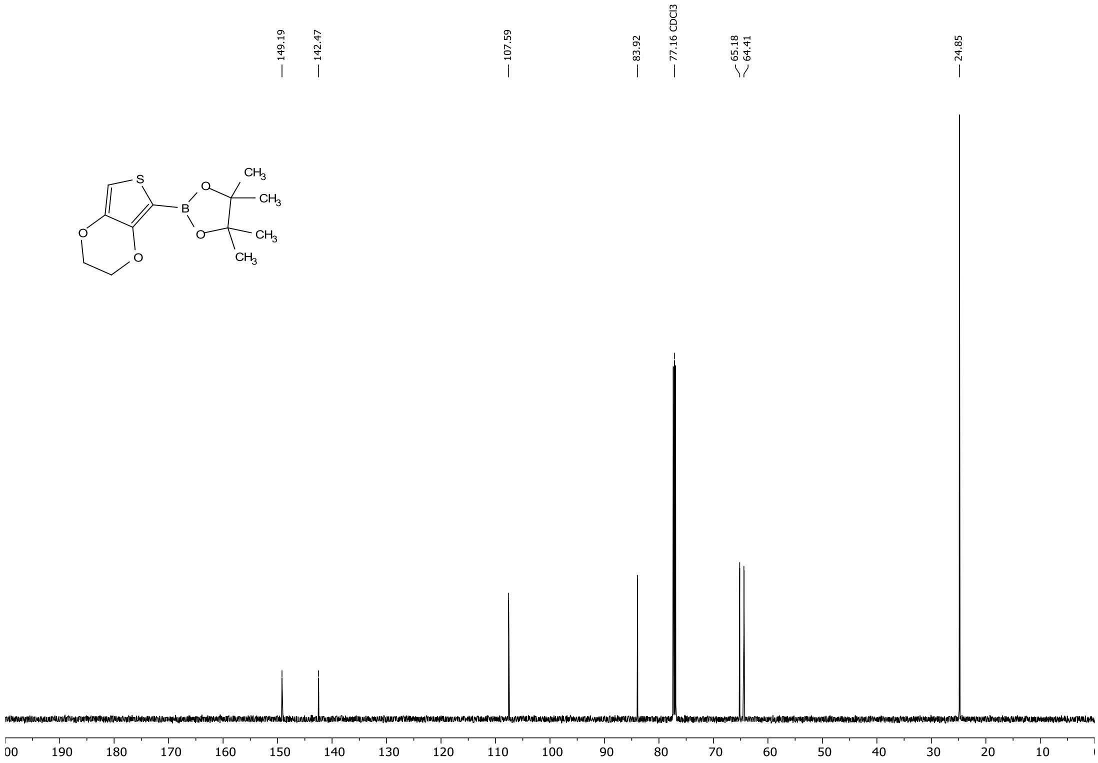

(b)

Figure S1: (a) ^{1}H-NMR (600 MHz, $CDCl_3$) of compound **2**. (b) ^{13}C{H}-NMR (151 MHz, $CDCl_3$) of compound **2**.

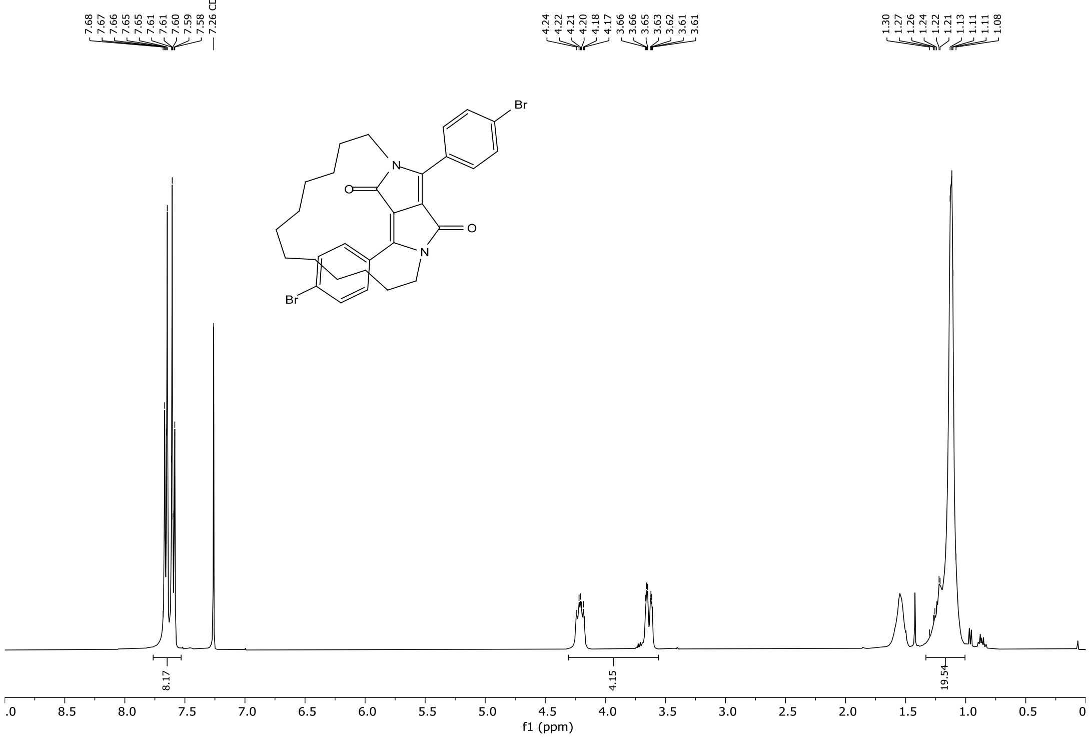

(a)

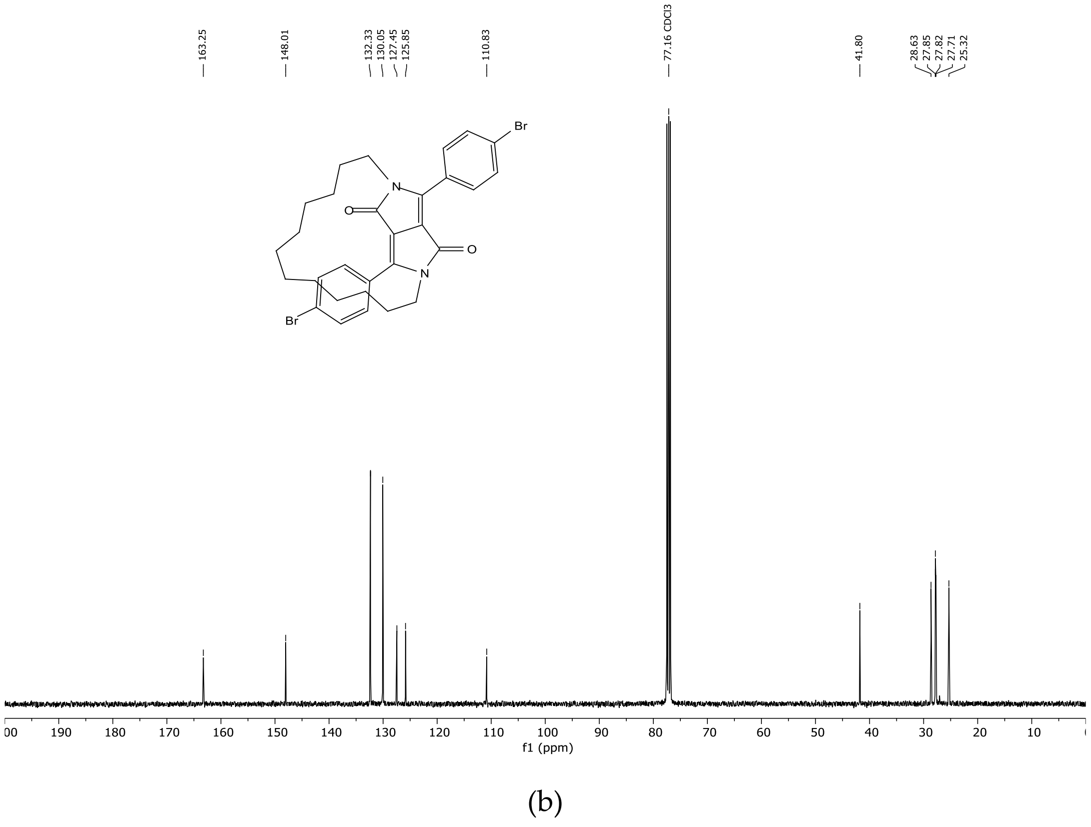

(b)

Figure S2: (a) ^{1}H-NMR (400 MHz, $CDCl_3$) of **(*rac*)-Br-DPP**. (b) ^{13}C{H}-NMR (101 MHz, $CDCl_3$) of **(*rac*)-Br-DPP**.

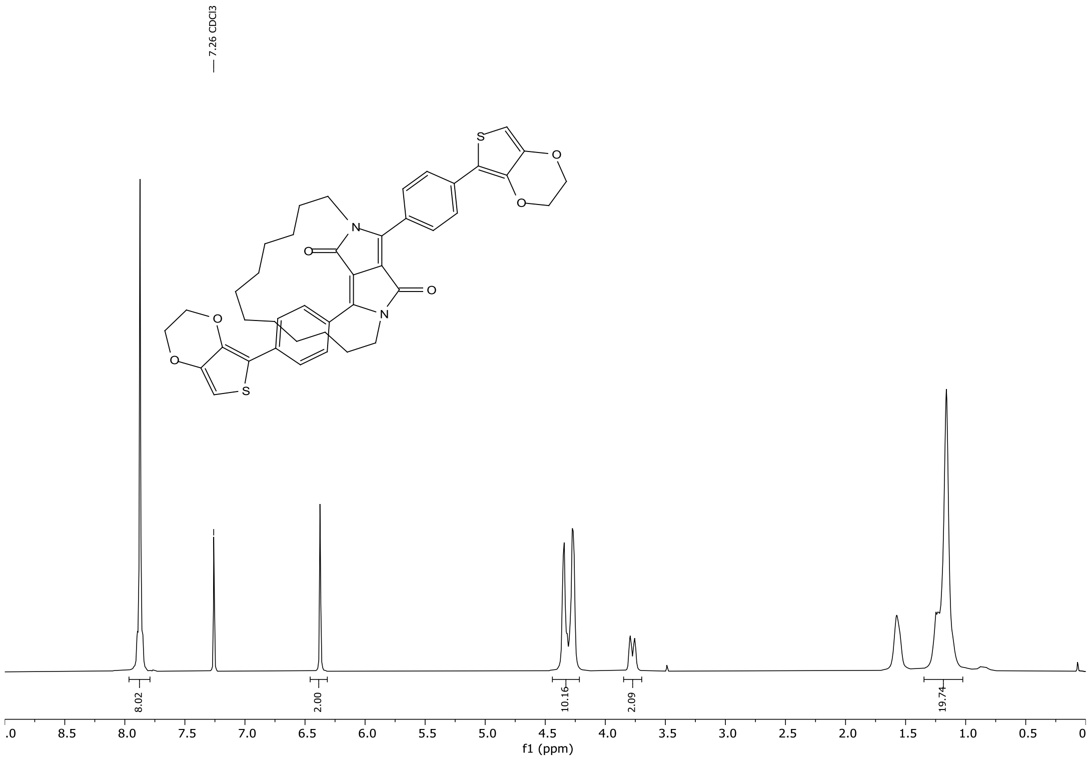

(a)

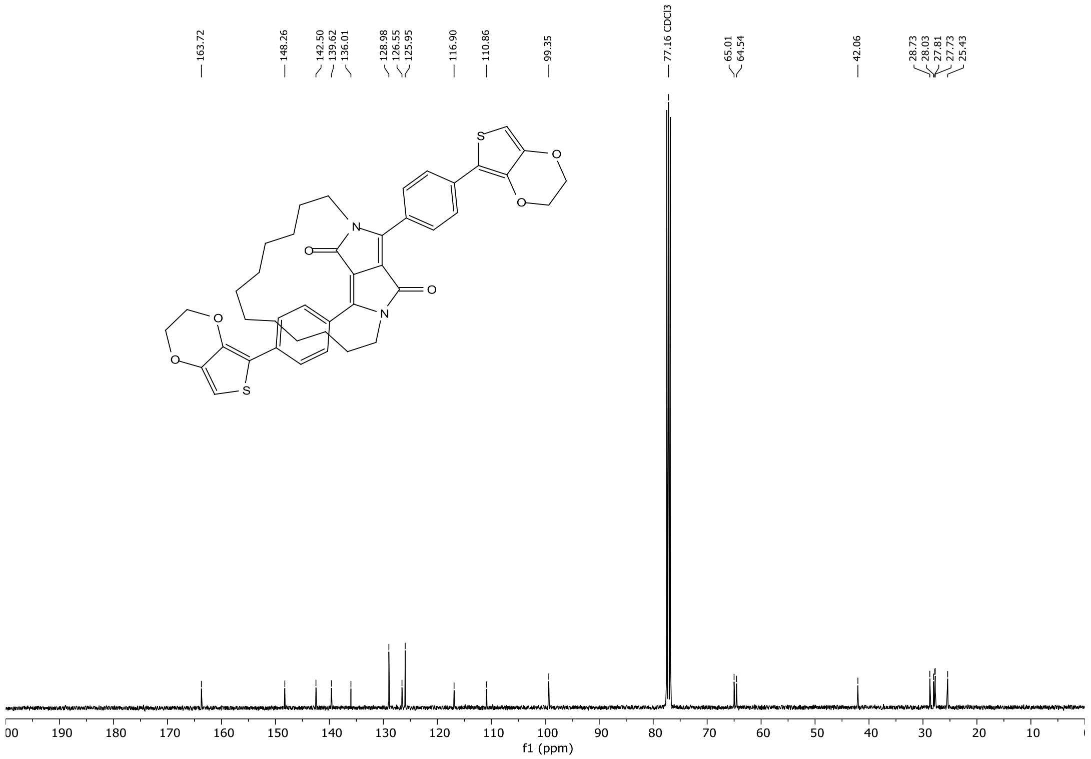

(b)

Figure S3: (a) ^{1}H-NMR (400 MHz, $CDCl_3$) of **(*rac*)-EDOT-DPP-EDOT**. (b) ^{13}C{H}-NMR (101 MHz, $CDCl_3$) of **(*rac*)-EDOT-DPP-EDOT**.

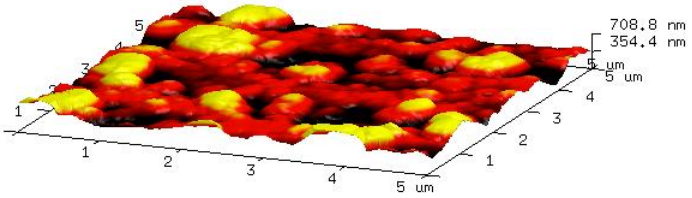

(a)

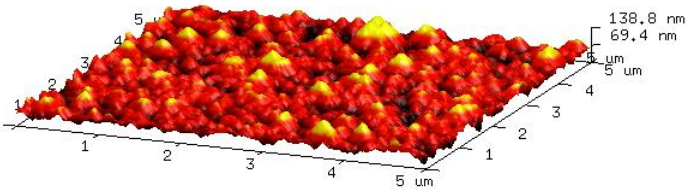

(b)

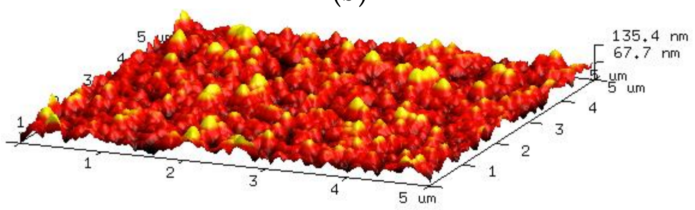

(c)

Figure S4: Tapping mode AFM 3-D-images of polymer films on ITO. (a) **P-*(rac)*-EDOT-DPP-EDOT**, (b) **P-*(–)*-EDOT-DPP-EDOT**, (c) **P-*(+)*-EDOT-DPP-EDOT**.

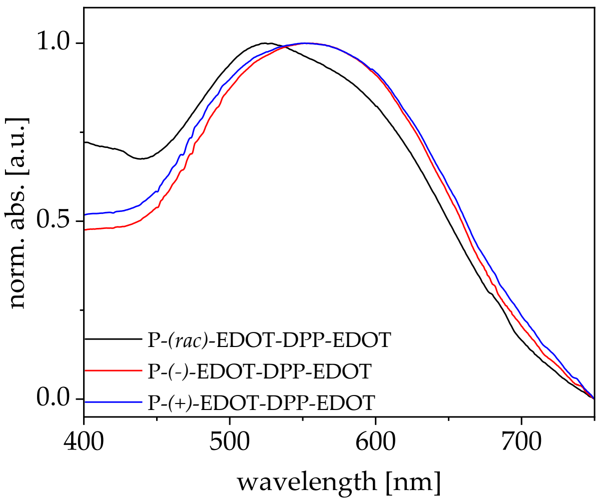

Figure S5: UV/Vis spectra of polymer films measured on ITO. **P-*(rac)*-EDOT-DPP-EDOT** measured on a JASCO V-670 spectrometer, **P-*(–)*-EDOT-DPP-EDOT,** and **P-*(+)*-EDOT-DPP-EDOT** measured on a Jasco J-810 CD- spectropolarimeter.
